# Fundamental frequency measures in the comparison of habitual and disguised voices

**DOI:** 10.1590/2317-1782/e20250251en

**Published:** 2026-05-11

**Authors:** Amanda de Castro Matheus, Denise de Oliveira Carneiro Berejuk, Eliane Cristina Pereira, Perla do Nascimento Martins, Ana Paula Dassie-Leite

**Affiliations:** 1 Departamento de Fonoaudiologia, Universidade Estadual do Centro-Oeste – UNICENTRO - Irati (PR), Brasil.; 2 Polícia Científica do Paraná - Curitiba (PR), Brasil.; 3 Colegiado de Licenciatura em Teatro, Universidade Estadual do Paraná – UNESPAR - Curitiba (PR), Brasil.

**Keywords:** Voice, Voice Quality, Speech Acoustics, Speaker Recognition Interface, Acoustics

## Abstract

**Purpose:**

This study aimed to investigate the stability of measures of fundamental frequency of voice by comparing samples of habitual and disguised speech.

**Methods:**

This observational, analytical, cross-sectional study analyzed a voice bank of 40 people of both sexes (20 men and 20 women), aged 19 to 58 years. They read a passage in both habitual and disguised speech. The types of disguises were analyzed by three expert voice judges. The study used the PRAAT^®^ acoustic analysis software to extract the mean f_0_, median f_0_, minimum f_0_, maximum f_0_, and base f_0_ (f_b_) from the complete reading in both situations, using pre-determined procedures. The data were tabulated and statistically analyzed.

**Results:**

The f_b_, maximum f_0_, and minimum f_0_ values were similar in habitual and disguised speech among speakers of both sexes, whereas the mean f_0_ and median f_0_ differed between the two situations.

**Conclusion:**

In general, the measures of f_b_, minimum f_0_, and maximum f_0_ were more stable in disguised speech, which may suggest future usefulness in intraindividual comparisons. The speakers’ various types of adjustments to disguise their voices did not significantly alter the measures of f_b_, maximum f_0_, and minimum f_0_ in the comparison between habitual and disguised speech.

## INTRODUCTION

Forensic speaker comparison (FSC) is an examination indicated in cases where it is necessary to determine the authorship of speeches possibly linked to a crime that is recorded on media^(1)^, comparing stored speeches with the suspect’s speech records. This comparison between materials makes it possible to arrive at a result that confirms or refutes the hypothesis of authorship. In most cases, the traces used in the forensic examination come from audio recordings originating from fixed or mobile telephone interceptions, environmental recordings made and authorized by the courts, audios recorded by one of the interlocutors in telephone communication, and from messaging applications (for example, WhatsApp)^(1)^.

Acoustic analysis is fundamental in speaker comparison and a premise of international consensus, as it considers quantitative aspects and the possible visual association. The former includes objective values ​​of various parameters, which can be analyzed both within and between speakers^(2,3)^, whereas visual association is made possible through broadband and narrowband spectrograms and lends credibility to the analysis^(4-8)^. Acoustic analysis is an important benchmark in comparing speakers, as it allows for greater understanding and comparison of the vocal signal, associations with auditory-perceptual evaluation, and visual documentation of voice and speech parameters^(9)^.

Fundamental frequency of the voice (f_0_) is one of the acoustic measures frequently used in FSC^(10-14)^, corresponding to the number of complete cycles of vocal fold opening and closing^(9)^. Research carried out at the University of York^(9)^ indicated that f_0_ is the most used acoustic measure in FSC and that 94% reported using arithmetic mean measures, 72% standard deviation, 41% median, 34% mode, 25% base f_0_ (fb), and 6% reported analyzing the range of f_0_ values. The widespread use of f_0_ in this examination is explained by its qualities and for being measurable and available within a speech sample^(10)^.

F_0_, the number of vibrational cycles of the vocal folds per second, has a physiological characteristic related to vocal fold length, which can produce population averages^(11)^. This frequency is the best known and most used parameter when it comes to voice recognition and speaker comparison^(12-16)^. Moreover, the most frequent forms of disguise directly affect f_0_^(11)^, which indicates frequency variations in the voice, such as high and low pitch^(15)^.

The speech style, vocal effort, and emotional state can affect f_0_, generating intra-speaker variation and decreasing its discriminative power^(1)^. A study reported that only 25% of those consulted use the long-term measure of f_0_ (LT f_0_) – i.e., f_b_ values^(9)^. However, recent research has indicated that this measure seems to be more stable than other f_0_ parameters^(16,17)^.

F_b_, proposed by Swedish phoneticians Traunmüller and Eriksson^(18)^, is a specific statistical estimator of location for f_0_ samples. F_b_ is the frequency of vibration of the vocal folds in a relaxed position, the frequency to which the vocal folds naturally return after a prosodic excursion. According to modulation theory, there must be a base value of the fundamental frequency, whose frequency represents the individual articulation of the speaker. Hence, it would be the best predictor of the individual's intrinsic f_0_ value, corresponding to the personal frequency of the vocal folds, the neutral f_0_, characteristic of the speaker^(19)^.

The results of a recent study demonstrated that f_b_ is the best parameter to be used in FSC f_0_ analyses^(1)^. According to the authors, the statistical measure of the f_0_ baseline value is less affected by the speech style and content or the channel used in the recording and requires less audio to obtain a stable measure than commonly used f_0_ measures, including the arithmetic mean and standard deviation.

Studies are scarce in the literature on the analysis of voice disguise in forensic phonetics^(11,15,20,21)^. Nevertheless, they can be very useful for understanding evidence-based speaker comparison.

Considering that speaker comparison often encounters disguise^(22-24)^, it is necessary to understand how such comparisons are carried out. Giller^(25)^ defines disguise as “[…] the deliberate action of a speaker who alters their voice, speech, or language to conceal their identity”. From this definition, we can understand that disguise is an imitation of a speech style or accent to hinder comparisons of that subject’s voice. In forensics, disguise appears most often in cases of kidnapping, extortion, harassment, prank calls, drug trafficking operations, homicides, corruption, and fraud. Voice disguise in a possible crime is one of the factors that most hinders forensic analysis^(25)^.

Disguise can occur at the moment of the “questioned recording,” which is the term used in forensics for compared recordings. It can happen when the act occurs or when the subject knows that they are being recorded and will be subjected to comparison examination, this being a moment used for comparison between records^(4)^.

For forensic work, it is of paramount importance to seek a parameter that is little influenced by environmental conditions and disguise, since criminals seek strategies and resources that can camouflage their true identity in the face of future evidence.

A key point regarding voice disguise is that it is not self-exhausted – i.e., it can provide important information about the acoustic parameters and their behavior in disguise. Thus, when the speaker disguises their voice, it can be compared with their habitual voice^(26)^. Therefore, the subject’s habitual voice must be known in order to analyze their disguised voice^(11)^.

Given the growing demand for specialists in forensics, speech-language-hearing pathology uses knowledge applied to human communication in forensic science to deal with evidence left by a crime or an alleged crime. Forensic comparative analyses of the voice are among the various possible contributions of speech-language-hearing pathology to the field^(4)^.

This research is relevant in that it contributes to greater clarification and elucidation on the possibility of using acoustic measures of disguised voices in speech, since the literature still has gaps on the subject. It is estimated that by highlighting the best measures of speech for the comparison of disguised voices, it will be possible to use them more assertively, as they are easily extracted in any environment^(11,27,28)^.

Hence, this study aimed to investigate the stability of f_0_ measures of the voice, comparing samples of habitual and disguised speech.

## METHODS

This is an observational, analytical, cross-sectional study. The analysis sample was obtained from a voice bank collected for a previous study. The research was approved by the Research Ethics Committee of the Universidade Estadual do Centro-Oeste, UNICENTRO, Paraná, Brazil, under number 4.752.583.

### Sample

A database of speech samples from 40 people was analyzed, subdivided according to sex and age as follows: 20 women, aged 19 to 55 years (average age: 32 years and 3 months), and 20 men, aged 19 to 55 years (average age: 33 years and 4 months). The group consisted of university students, university professors, and experts, with no professional announcers.

The voice bank samples were collected in a laboratory with acoustic treatment on a Pentium Dual Core 5.300 2.60 GHz computer, 1.99 GB RAM, XP 2002 Service Pack 3 processor, M-Audio Fast Track Pro 4x4 external sound card, and AKG C 3000 B microphone.

The audio recording was collected in wave format, mono channel, sampling frequency of 44.1 kHz, and 16 bits. The duration of the samples varied according to each speaker’s reading/speaking speed, ranging from 0.40 seconds to 2 minutes per audio.

The recordings were made using the Audacity^®^ program. The voices were collected inside an acoustic booth, where the microphone was located. For the recording, the speakers were instructed neither to vary their body position to avoid distancing from the microphone nor to incline their neck to avoid modifications in laryngeal movement.

The speech text collected and used in this research simulated a distress call during a phone call and was read by participants four times: twice with their usual voice and twice with a disguised voice. For disguised reading, they were instructed to try not to be recognized, using any resources they deemed necessary. Thus, speakers were free to choose a disguise they considered effective, as long as they did not use resources other than their articulatory organs (e.g., using their hands to occlude the nostrils or any other type of interference).

Habitual speech was collected twice, so that the subject could become familiar with the text. This research used the second speech recording, considered the habitual speech sample, and the first disguised recording because speakers generally used similar resources in both, not considering whether they could maintain the disguise.

Previous studies had already used the voice bank. Researchers in literature/linguistics and forensics developed the following text (consisting of 69 words in Portuguese) during a master's thesis: "Hello, I want to speak with Dona Teca. Dona Teca, this is the devil speaking. We have your husband's duck in the den, and we're going to kill him, chop him up, and throw him in a Coke bottle. Do you want to save him? Then don't make me angry. I want a million. Put everything in a package near the peccary's cask and run away. Don't call the police or I'll poke you and stab your head" (in Portuguese: *"Alô, quero falar com a Dona Teca. Dona Teca, aqui fala é o capeta. Estamos com o pato do teu marido na toca e vamos matar ele, picar e tacar dentro de uma garrafa de Coca. Quer salvar ele? Então não me deixa puto. Quero um milhão. Bota tudo num pacote perto da pipa do cateto e se pica. Não chama a polícia senão te cutuco e espeto teu coco"*).

Its use in disguise considered various phonemes, especially intervocalic voiceless plosives [p, t, k], the onset of stressed syllables, the phonological context preceding the vowel [a], and the phonological context following [p]: [e, i, u]; following [t]: [a, e, ɛ, ɔ], and following [k]: [a, ɔ].

### Disguise analysis

Three judges, experts in voice, with more than 15 years of experience in auditory-perceptual evaluation, including protocols involving the analysis of vocal tract adjustments, such as the John Laver Vocal-Profile Analysis Protocol (1980), were invited to analyze the types of disguises. The analyses proceeded as follows: initially, two of the three judges analyzed the samples of each subject, in pairs, individually, considering the subject's habitual voice as a baseline. Thus, the judges had to point out which resources/adjustments the speaker used to disguise their voice in comparison with their habitual voice.

The researchers developed a questionnaire containing 21 possible types of adjustments, based on common adaptations made by people in disguise speech, generally related to modifications in voice quality, frequency, intensity, articulation, resonance, and modulation. The judges were to mark an “x” when the speaker used the characteristic to disguise their voice.

The responses that differed between the two judges were sent to the third judge for tie-breaking and confirmation of whether the speaker used that adjustment. Moreover, 20% of the samples were repeated for intrarater agreement analysis. The kappa value obtained was greater than 0.8 for all of them, indicating substantial intrarater agreement.

The PRAAT^®^ acoustic analysis software was used to extract f_0_ data, adopting the following steps for all samples from each participant (habitual and disguised): Importing the complete reading excerpt; analyzing the file's periodicity in relation to the pitch (f_0_), with adjustment of the parameters according to the speaker's sex; generating a specific file for f_0_ analysis; visually analyzing the f_0_ points of the generated file to eliminate spurious points and artifacts; extracting f_0_ measures from the corrected file: mean (sum of the values ​​obtained in the set throughout the entire section, divided by the number of values ​​summed), median (value that separates the larger half from the smaller half of a sample), f_b_ (statistical measure that defines the frequency to which the vocal folds naturally return after a prosodic excursion)^(18,19)^, minimum (lowest f_0_ value observed throughout a section) and maximum (highest f_0_ value observed throughout a section); and creating a distribution graph from the pitch file, verifying the f_0_ distribution.

Thus, the extraction followed these steps: 1. Adjusting the pitch analysis limits according to sex (75 to 300 Hz in males and 100 to 500 Hz in females); 2. Verifying the behavior of the pitch curve generated with the pulses considered automatically; 3. Not considering the production times of the segments of each individual, given that the calculations of f_0_ and f_b_ are measured in Hz; 4. Creating pitch object; 5. Visually comparing the oscillogram, pulses, and pitch curve of the sound object with the points (samples) of the pitch object, excluding samples that did not correspond to a voice signal; 6. Obtaining the frequency values ​​of the corrected pitch object; 7. Creating the distribution graph from the pitch object matrix.

The descriptive analysis of the results of the quantitative variables was performed using relative and absolute frequency. All numerical values ​​obtained in the procedures described above were tabulated in a spreadsheet.

The mean, median, minimum, and maximum f_0_ values ​​and f_b_ values ​​of habitual voices were compared with the mean, median, minimum, and maximum f_0_ values ​​and f_b_ values ​​of disguised voices using statistical tests for dependent samples. Thus, the habitual speech sample was considered the control sample in relation to the disguised speech sample.

### Statistical analysis

The study used Student's dependent t-test and the Wilcoxon test. The dependent sample tests were also used to compare the mean, median, minimum, and maximum f_b_ during habitual and disguised speech, for men and women. Student's dependent t-test or Wilcoxon test was used, depending on the data distribution (normal or abnormal), which was analyzed with the Shapiro-Wilk normality test. In the case of statistically significant differences, the effect size was calculated using Cohen's d (variables with differences had a normal distribution). The interpretation of the effect size was as follows: 0.20 small effect, 0.5 medium effect, and 0.8 large effect.

All analyses used a 5% significance level, or 0.05.

## RESULTS

Table 1 presents the characterization of the sample through descriptive analysis of the speakers’ vocal adjustments when disguising their voices in relation to their usual voices (Table 1).

**Table 1:** Descriptive analysis of the types of disguises used by speakers

**Variable**	**Total sample (n=40)**		**Females (n=20)**		**Males (n=20)**	
	**n**	**%**	**n**	**%**	**n**	**%**
Strained voice	21	52.50	9	45.00	12	30.00
Greater loudness	21	52.50	10	50.00	11	27.50
Lower pitch	18	45.00	9	45.00	9	45.00
phoneme distortions/ omissions/substitutions	16	40.00	5	25.00	11	27.50
Higher pitch	14	35.00	9	45.00	5	25.00
Pharyngeal constriction	14	35.00	4	20.00	10	50.00
Roughness/crepitation	12	30.00	3	15.00	9	45.00
Lip rounding/protrusion	8	20.00	5	25.00	3	15.00
Articulatory/jaw closure/locking	6	15.00	2	10.00	4	20.00
Hypernasality	6	15.00	2	10.00	4	20.00
Lip stretching	5	12.50	2	10.00	3	15.00
Falsetto	4	10.00	1	5.00	2	10.00
Pitch variations	3	7.50	3	15.00	0	0.00
Loudness variations	3	7.50	0	0.00	0	0.00
Lower loudness	2	5.00	1	5.00	1	5.00
Hyponasality	2	5.00	2	10.00	0	0.00
Excessive jaw opening/ overarticulation	2	5.00	1	5.00	1	5.00
Whisper/ whispered voice	1	2.50	1	5.00	0	0.00
Vocal tremor	1	2.50	0	0.00	1	5.00
Breathiness	0	0.00	0	0.00	1	5.00
Relaxed voice	0	0.00	0	0.00	0	0.00

Strained voice and greater loudness were the most used (n = 21; 52.50%), followed by lower pitch (n = 18; 45%), phoneme distortions/omissions/substitutions (n = 16; 40%), higher pitch, and pharyngeal constriction (n = 14; 35% in both). Although there were several higher relative frequencies in descriptive terms between men and women, the equality of proportions test, performed complementarily, indicated p > 0.05 in 20 of the 21 cross-matches between the sexes, with a difference only for pharyngeal constriction, which men used (n = 10; 50%) more than women (n = 4;20%) – p = 0.047.

The comparison between f_0_ results in disguised and habitual voices showed no differences in f_b_, minimum f_0_, or maximum f_0_. The same was not observed for the mean f_0_ (habitual 198.85 and disguised 216.62 – p = 0.021) or median f_0_ (habitual 195.31 and disguised 206.1 – p = 0.032), which were different between emissions, with higher values ​​in disguised speech (Table 2). The additional calculation of Cohen's d indicated a small to medium effect for the mean f_0_ and median f_0_.

**Table 2:** Comparison between the results of the fundamental frequency of the voice (f_0_) during habitual and disguised speech in the total group (n = 40)

Variable	Habitual speech (H)					Disguised speech (D)					p	Effect size
	Mean	Median	Minimum	Maximum	SD	Mean	Median	Minimum	Maximum	SD		
Base f_0_^***^	147.276	141.422	86.684	214.887	39.633	153.42	158.533	83.532	250.333	44.356	0.333	
Mean f_0_^**^	198.857	200.22	109.132	299.64	56.289	216.624	210.061	120.428	365.543	59.607	**0.021** ^*^	0.38
Median f_0_**	195.31	198.842	106.322	286.391	56.09	211.87	206.106	112.624	367.332	62.018	0.032*	0.35
Minimum f_0_***	79.584	76.349	50.315	121.211	13.602	80.581	75.804	53.351	158.172	20.272	0.751	
Maximum f_0_***	485.904	579.378	179.868	639.778	144.06	486.715	553.68	195.081	609.516	126.846	0.968	

* p < 0.05

** Student's dependent t-test and effect size with Cohen’s d

*** Wilcoxon test

Caption: SD = standard deviation

The comparison between f_0_ results in disguised and habitual voices in females found no differences in any of the measures analyzed (Table 3).

**Table 3:** Comparison between the results of the fundamental frequency of the voice (f_0_) during habitual and disguised speech among females (n = 20)

Variable	Habitual speech (H)					Disguised speech (D)					p
	Mean	Median	Minimum	Maximum	SD	Mean	Median	Minimum	Maximum	SD	
Base f_0_^***^	182.909	182.017	143.504	214.887	17.456	183.043	182.707	107.446	250.333	31.343	0.709
Mean f_0_^**^	248.492	248.805	201.112	299.64	28.463	250.495	236.291	188.194	365.543	46.565	0.808
Median f_0_**	244.347	243.453	199.114	286.391	28.914	247.385	234.939	183.036	367.332	47.504	0.729
Minimum f_0_***	82.325	78.949	64.471	121.211	14.163	83.703	75.825	53.351	158.172	27.305	0.970
Maximum f_0_***	531.226	584.084	316.243	639.778	101.149	512.296	570.637	314.665	609.516	102.26	0.433

** Student’s dependent t-test

*** Wilcoxon test

Caption: SD = standard deviation

Finally, Table 4 shows the comparison between f_0_ results in habitual and disguised speech in males. This analysis found differences between the mean f_0_ (habitual 149.22 and disguised 182.75 – p = 0.09) and median f_0_ (habitual 146.27 and disguised 176.35 – p = 0.018). The table shows medium to large effect sizes of the mean f_0_ and median f_0_.

**Table 4:** Comparison between the results of the fundamental frequency of the voice (f_0_) during habitual and disguised speech in males (n = 20)

Variable	Habitual speech (H)					Disguised speech (D)					p	Effect size
	Mean	Median	Minimum	Maximum	SD	Mean	Median	Minimum	Maximum	SD		
Base f_0_^***^	111.643	114.4	86.684	139.339	15.697	123.796	118.027	83.532	228.158	34.765	0.108	
Mean f_0_^**^	149.222	148.514	109.132	199.329	22.516	182.753	172.251	120.428	314.233	52.053	**0.009** ^*^	0.66
Median f_0_**	146.274	145.35	106.322	198.57	23.655	176.355	158.664	112.624	331.095	54.616	0.018*	0.58
Minimum f_0_***	76.844	75.484	50.315	96.274	12.781	77.458	75.804	65.208	102.531	8.799	0.737	
Maximum f_0_***	440.582	525.051	179.868	601.281	167.461	461.134	532.108	195.082	602.068	145.574	0.332	

* p < 0.05

** Student’s dependent t-test and effect size with Cohen’s d

*** Wilcoxon test

Caption: SD = standard deviation

## DISCUSSION

Pitch changes during speech, going lower and higher, as it is the element responsible for intonation (frequency variation from the point of view of psychoacoustic perception)^(15)^. Speakers vary their pitch because of the various tones used to express themselves. Hence, the f_0_ acoustic analysis allows the plotting of graphs called pitch contour curves to find instantaneous f_0_ values as a function of time^(29)^.

The results of this study reinforce a previous study^(15)^ regarding the common pitch variations in disguise speech, in that the lower pitch was the second most used resource, appearing in 45% of the disguises of the general, female, and male populations. The higher pitch also appeared among the speakers’ preferred disguises, with 32.5% of the general population. Pitch variations occurred in 7.5% of the disguise cases in this study in the general group, appearing only in the female population, in 15% of the disguises.

The authors of a recent study^(30)^ analyzed the effects of disguise on the f_0_ and whether these affected the acoustic analyses. They observed that one of the most used types of adjustments was tract constriction, strongly associated with disguise f_0_ values in the control samples of men (p = 0.105) and women (p = 0.171). Thus, it can be understood that even with tract constriction, it is possible to perform acoustic analyses and ascertain whether the same subject is speaking.

Another type of disguise present in the study is the participant’s simulation of anger^(30)^. The variations that anger can bring to disguise speech also appeared in this study, translating as greater loudness, which was the resource most used by the total group. All the resources regarding the previous study^(30)^ were strongly associated with the disguise f_0_ values of the control of men and women samples – females had greater variations in disguise f_0_ values than males, a result that is divergent from the present study, in which women made smaller changes in f_0_ than men.

The opinion of experts has been increasingly requested and included in legal processes to answer whether a given voice sample belongs to the same speaker^(3)^. This sample comparison and its results correspond to what is called forensic identification of the speaker^(4)^. This study verified that some acoustic measures are more stable in the comparison between habitual and disguise speech samples of the same individual, which could be confirmed by the lack of statistically significant differences. This is evidenced by the similar f_b_, maximum f_0_, and minimum f_0_ results in both samples despite the speakers’ various vocal tract adjustments. These results indicate that such measures are not susceptible to change caused by vocal tract modifications, which may contribute to the expert report, confirming or refuting the hypothesis that it is the same speaker in the possible crime in question (Table 2).

These results indicate that f_b_, maximum f_0_, and minimum f_0_ change less than the other frequencies. This draws a parallel with another study^(11)^, whose statistical analyses demonstrated differences in intra-speaker mean and median f_0_ when different types of disguises were simulated by varying speech style, emotions, vocal effort, and recording quality. Study authors^(16)^ report that the results of mean f_0_, median f_0_, and standard deviation were affected by outliers – a term used in statistics to refer to atypical findings that deviate from normal, possibly harming the interpretation of the results of the samples in question. As for the present study, such outliers were removed to minimize this bias.

This study obtained similar f_b_, maximum f_0_, and minimum f_0_ results in habitual and disguised speech, despite the varied resources used by speakers. F_b_ is the frequency of vocal cord vibration in a relaxed position, the frequency to which the vocal folds naturally return after a prosodic excursion^(19)^. Thus, the hypothesis is that it is less susceptible to diverse speech variations from a prosodic standpoint and from articulatory organ modifications, which can contribute to the expert report when comparing speakers in crime analysis.

To date, forensic f_0_ analysis has focused on its mean duration and standard deviation^(15)^. The analysis of the present study results suggests that the mean and median duration may be less stable measures for disguised speech and sample comparison, as they present statistically significant changes. External factors, such as susceptible emotions, may greatly influence the mean f_0_^(19)^. Perhaps this could explain why the mean and median f_0_ differed between habitual and disguised speech. It is important to mention that the overall group had small to medium effect sizes in the comparison of the mean and median f_0_ in habitual and disguised speech – i.e., they are visible effects that may be relevant in clinical and/or forensic contexts. However, these same measures had medium to large effect sizes in males, suggesting that the differences found have significant practical relevance.

Several factors, didactically divided into technical, physiological, and psychological factors, influence and affect f_0_. Physiological factors are biological causes, such as age and smoking, while psychological factors are linked to emotions and environmental conditions at the time of emission. Technical factors, in turn, refer to the sample recording conditions^(31)^. Nevertheless, f_0_ is a phonetic parameter with good results, being, therefore, considered reliable for forensic analysis^(10)^. Stability in speaker comparison parameters is a key resource in forensic expertise; hence, f_0_ seems to meet a wide range of these criteria^(31)^. Having f_0_ stability in mind, this study approached other f_0_ measures, strengthening the idea mentioned earlier^(16,17)^ that f_b_ is more stable than other f_0_ parameters, such as the already mentioned mean f_0_ and median f_0_.

This knowledge demonstrates that the expert comparison of disguised speech can use f_b_ along with the maximum and minimum f_0_, which have yielded reliable results in forensic analysis in cases of crimes or suspected crimes.

Regarding limitations, this study did not evaluate the natural f_0_ variability of the same speaker in undisguised speech. This could contribute to assessing how much the effects observed in this study were attributable to vocal disguise or fluctuations in vocal emission. Future studies are encouraged to approach this analysis.

Further research could use samples of spontaneous or semi-spontaneous phrases and speech, along with habitual and disguised text reading, to obtain estimate/cutoff values in Hz from one measure to another. Thus, it would be possible to establish an acceptable Hz parameter in the future to determine whether the samples are believed to be from the same subject, making more in-depth inferences.

Studies whose methodological designs include accuracy, sensitivity, and specificity measures may enable the differentiation between individuals and speakers, in addition to the intra-individual comparisons carried out in this research.

## CONCLUSION

The study results lead to the conclusion that f_b_, minimum f_0_, and maximum f_0_ were more stable in disguised speech, suggesting future usefulness in intra-individual comparisons. Hence, f_b_, a measure still little explored and disseminated in criminal forensics, proves useful for the comparative analysis of speakers, assisting in the production of the expert report. The speakers’ various types of adjustments to disguise their voices did not significantly alter f_b_, maximum f_0_, and minimum f_0_ measures in the comparison between habitual and disguised speech.
