# Supplementary material for: Fundamental frequency measures in the comparison of habitual and disguised voices
Source: Codas. 2026 May 11;38(4):e20250251. doi: 10.1590/2317-1782/e20250251en (PMC13193189; doi:10.1590/2317-1782/e20250251en)
Supplement: Translation

# Translation: Medidas de frequência fundamental na comparação de vozes habituais e em situação de disfarce

**DOI:** 10.1590/2317-1782/e20250251pt

**Authors:** Amanda de Castro Matheus, Denise de Oliveira Carneiro Berejuk, Eliane Cristina Pereira, Perla do Nascimento Martins, Ana Paula Dassie-Leite

**Affiliations:** 1 Departamento de Fonoaudiologia, Universidade Estadual do Centro-Oeste – UNICENTRO - Irati (PR), Brasil.; 2 Polícia Científica do Paraná - Curitiba (PR), Brasil.; 3 Colegiado de Licenciatura em Teatro, Universidade Estadual do Paraná – UNESPAR - Curitiba (PR), Brasil.

**Keywords:** Voz, Qualidade da Voz, Acústica da Fala, Interface para o Reconhecimento da Fala, Acústica

## Abstract

**Objetivo:**

Este estudo teve o objetivo de investigar a estabilidade das medidas de frequência fundamental da voz, comparando amostras de fala habitual e de fala em situação de disfarce.

**Método:**

Trata-se de estudo observacional, analítico e transversal. Foi analisado um banco de vozes de 40 pessoas de ambos os sexos (20 homens e 20 mulheres) e faixa etária entre 19 e 58 anos, correspondentes à leitura de trecho em fala habitual e leitura do mesmo trecho em fala em situação de disfarce. Os tipos de disfarces foram analisados por três juízas especialistas em voz. Além disso, foi utilizado software de análise acústica PRAAT® para a extração das medidas acústicas de f_0_ média, f_0_ mediana, f_0_ mínima, f_0_ máxima e f_0_ de base (f_b_) da leitura na íntegra, em ambas as situações, por meio de procedimentos pré-determinados. Os dados foram tabulados e analisados estatisticamente.

**Resultados:**

Os valores das medidas de f_b_, f_0_ máxima e f_0_ mínima foram semelhantes nas situações de fala habitual e disfarce, para locutores de ambos os sexos. Já os valores das medidas de f_0_ média e f_0_ mediana tiveram diferença nas duas situações.

**Conclusão:**

De modo geral, as medidas de f_b_, f_0_ mínima e f_0_ máxima mostraram maior estabilidade frente à situação de disfarce, o que pode sugerir utilidade futura em comparações intraindividuais. Os variados tipos de ajustes feitos pelos locutores para a realização dos disfarces não alteraram de modo significativo as medidas de f_b_, f_0_ máxima e f_0_ mínima quando as situações de fala habitual e disfarçada foram comparadas.

## INTRODUÇÃO

O exame de Comparação Forense de Locutor (CFL) é indicado nos casos em que é preciso apontar a autoria de falas possivelmente ligadas a um crime que esteja gravado em mídia)^(1)^. Para isso, as falas armazenadas são submetidas à comparação entre os registros de fala do suspeito. Por meio do confronto entre os materiais pode-se chegar ao resultado que confirme ou refute a hipótese da autoria. Em sua maioria, os vestígios utilizados no exame forense são provenientes de áudios oriundos de interceptações telefônicas fixa ou móvel, gravações ambientais realizadas e autorizadas pela justiça, áudios gravados por um dos interlocutores na comunicação telefônica e de aplicativos de mensagens (por exemplo, Whatsapp)^(1)^.

A análise acústica é fundamental no processo de comparação de falantes, sendo premissa de consenso internacional, pois leva-se em conta sua natureza quantitativa e ainda a possível associação visual. Como natureza quantitativa, temos a objetividade dos valores que se referem a diversos parâmetros, que podem ser analisados tanto inter quanto intrafalantes^(2,3)^. Já a associação visual é possibilitada através do espectrograma de banda larga e banda estreita e concedem credibilidade à análise^(4-8)^. A análise acústica é uma importante balizadora na comparação de locutores, pois ela possibilita a maior compreensão do sinal vocal, associações à análise perceptiva-auditiva, documentação visual dos parâmetros da voz e da fala, além de permitir a comparação^(9)^.

Uma das medidas acústicas frequentemente utilizadas no exame de CFL é a da frequência fundamental da voz (f_o_)^(10-14)^, que corresponde ao número de ciclos completos de abertura e fechamento das pregas vocais^(9)^. Uma pesquisa realizada na Universidade de York^(9)^ indicou que, dentre as medidas acústicas utilizadas no exame de CFL, a f_o_ é a mais frequentemente utilizada. No estudo supracitado sobre a f_o_, 94% referiram usar medidas de média aritmética, 72% de desvio padrão, 41% de mediana, 34% de moda, 25% de f_o_ de base (f_b_) e 6% relataram analisar o *range* dos valores de f_o_. O uso da f_o_ como medida disseminada nesse exame se explica pelas qualidades dessa medida, sendo ela considerada passível de mensurabilidade e disponibilidade dentro de uma amostra de fala^(10)^.

A frequência fundamental, número de ciclos vibratórios das pregas vocais em um único segundo, tem característica fisiológica relacionada ao comprimento das pregas vocais, passível de produção de médias populacionais^(11)^. Esta frequência se caracteriza como o parâmetro mais conhecido e mais usado quando se trata de reconhecimento de voz e comparação de falantes^(12-16)^. Além disso, as formas mais frequentes de disfarce afetam diretamente a frequência fundamental^(11)^. Na voz, a f_o_ indica as variações de frequência, como os sons graves e agudos^(15)^.

A f_o_ pode ser afetada pelo estilo de fala, pelo esforço vocal e pelo estado emocional, o que gera uma variação intrafalante, diminuindo seu poder discriminativo^(1)^. Estudo referiu que apenas 25% dos consultados utiliza a medida de longo termo da frequência fundamental (LT f_o_), isto é, o valor de f_b_^(9)^. No entanto, pesquisas recentes têm indicado que tal medida parece ser mais estável quando comparada a outros parâmetros de f_o_^(16,17)^.

O valor da f_b_, proposta pelos foneticistas suecos Traunmüller e Eriksson^(18)^, é um estimador estatístico de localização específico para amostras de f_o_. A f_b_ é a frequência de vibração das pregas vocais em uma posição relaxada, a frequência para qual as pregas vocais retornam naturalmente após uma excursão prosódica. De acordo com a teoria da modulação, deve existir um valor de base da frequência fundamental, cuja frequência representa a articulação individual do falante, sendo ele o melhor preditor do valor da f_o_ intrínseca do indivíduo, correspondendo à frequência pessoal das pregas vocais, o valor neutro da f_o_, característico do falante^(19)^.

Em estudo recentemente realizado, os resultados demonstraram que o valor de base de f_o_ é o melhor parâmetro a ser utilizado em análises de f_o_ em comparação Forense de Locutor^(1)^. Segundo os autores, a medida estatística valor de base de f_o_ é menos afetada pelo estilo e conteúdo da fala, pelo canal utilizado na gravação e exige menor quantidade de áudio para se obter uma medida estável, comparada às medidas de f_o_ comumente utilizadas, entre elas, a média aritmética e o desvio padrão.

Quanto à análise do disfarce de voz na fonética forense, há escassez de estudos na literatura^(11,15,20,21)^, e entende-se que eles podem ser muito úteis para a compreensão da comparação de falantes baseada em evidências.

Levando em consideração que a comparação de falantes muitas vezes se depara com o disfarce^(22-24)^, é preciso entender como tais comparações são realizadas. Giller^(25)^ define disfarce como sendo: “[...] *a ação deliberada de um falante que altera a sua voz, discurso ou língua com o propósito de esconder a sua identidade*”. A partir dessa definição conseguimos entender que o disfarce trata de uma imitação de um estilo de fala ou de um sotaque, que tem por objetivo dificultar a comparação da voz desse sujeito. No que diz respeito à área forense, o disfarce aparece com maior frequência em casos de sequestros, extorsões, assédio, trotes telefônicos, operações do narcotráfico, homicídios, corrupção e estelionato. A presença do disfarce num possível crime é um dos fatores que mais traz impasses à realização da análise forense^(25)^.

O disfarce pode acontecer no momento da “gravação questionada”, sendo este o termo usado na perícia para as gravações comparadas. Ele pode acontecer no momento que o ato ocorre, mas também quando o sujeito sabe que está sendo gravado e será submetido ao exame de comparação, sendo este um momento usado para confronto entre os registros^(4)^.

Para o trabalho pericial é de suma importância a busca por um parâmetro que seja pouco influenciável por condições ambientais e pelo disfarce, uma vez que os criminosos buscam por estratégias e recursos que possam camuflar sua verdadeira identidade diante de futuras provas.

Um ponto chave quando se refere ao disfarce de vozes é que o disfarce não é exaurido nele próprio, ou seja, ele pode vir a fornecer informações importantes sobre os parâmetros acústicos e seus comportamentos em situação de disfarce. Assim, quando o sujeito locutor se utiliza de disfarce na voz é possível realizar a comparação da voz habitual e com voz disfarçada^(26)^. Deste modo, para que se possa realizar análises acerca da voz disfarçada é preciso primeiro conhecer a voz habitual do sujeito em questão^(11)^.

Tendo em vista a crescente procura por especialistas na área forense, a Fonoaudiologia atua na Ciência Forense através do conhecimento aplicado à Comunicação Humana de forma a tratar de evidências deixadas por um crime ou por um suposto crime. Dentre as várias contribuições possíveis da Fonoaudiologia estão as análises de comparação forense da voz^(4)^.

A presente pesquisa mostra-se relevante a fim de contribuir para maior esclarecimento e elucidação sobre a possibilidade do uso das medidas acústicas de f_o_ em situações de vozes com disfarce já que a literatura ainda tem lacunas sobre a temática. Estima-se que ao serem evidenciadas quais as melhores medidas de f_o_ para a comparação de vozes disfarçadas, será possível atuar de forma mais assertiva na utilização dessas medidas, que são de fácil extração em quaisquer ambientes^(11,27,28)^.

Diante do exposto, o presente estudo teve o objetivo de investigar a estabilidade das medidas de frequência fundamental da voz, comparando amostras de fala habitual e de fala em situação de disfarce.

## MÉTODO

Trata-se de estudo observacional, analítico e transversal. A amostra da presente pesquisa analisou um banco de vozes coletado para pesquisa anterior. A pesquisa foi aprovada pelo Comitê de Ética em Pesquisa da Universidade Estadual do Centro-Oeste, UNICENTRO, sob o número 4.752.583.

### Amostra

Foi analisado um banco de dados composto por amostras de fala de 40 pessoas, subdivididas de acordo com o sexo e a idade da seguinte forma: 20 mulheres, com idades entre 19 e 55 anos (média de idade: 32 anos e 3 meses); 20 homens, com idades entre de 19 e 55 anos (média de idade: 33 anos e 4 meses). O grupo foi composto por estudantes universitários, professores universitários e peritos, não havendo locutores profissionais.

A coleta das amostras vocais do banco de vozes foi realizada em laboratório com tratamento acústico em Computador Pentium Dual Core 5.300 2.60 GHz, 1.99 Gb RAM Processador XP 2002 Service Pack 3, placa de som externa M-Audio Fast Track Pro 4x4, Microfone AKG C 3000 B.

O registro de áudio foi coletado em formato *wave,* mono canal, frequência de amostragem de 44,1 KHz e 16 bits. A duração das amostras variou de acordo com a velocidade de leitura/fala de cada locutor, tendo uma média de 0,40 segundos até 2 minutos de duração em cada áudio.

As gravações foram realizadas no programa Audacity®. As vozes foram coletadas no interior de uma cabine acústica, na qual se encontrava o microfone. Para a gravação, os locutores receberam orientações sobre não variar sua posição corpórea, para que não ocorresse distanciamento do microfone, como também inclinação cervical que pudesse modificar o movimento laríngeo.

O texto de fala coletado e utilizado na presente pesquisa simulava um pedido de resgate durante um telefonema e foi lido pelo informante quatro vezes: duas vezes com sua voz habitual e duas vezes com a voz disfarçada. A instrução dada para a leitura disfarçada foi de que o interlocutor buscasse não ser reconhecido, podendo ser utilizados quaisquer recursos que julgasse necessários. Assim, os falantes tiveram a liberdade de escolher um disfarce que considerassem eficiente, desde que não utilizassem recursos que extrapolassem seus próprios órgãos articulatórios, por exemplo, o uso das mãos não era permitido (para oclusão das narinas ou outro tipo de interferência).

As emissões habituais foram coletadas duas vezes a fim de que o sujeito se familiarizasse com o texto. Para a presente pesquisa foi utilizada a segunda emissão, sendo esta considerada a amostra de emissão habitual. No que diz respeito às emissões de disfarce, utilizou-se apenas a primeira emissão, já que, no geral, os locutores utilizaram recursos semelhantes em ambas e, também por não se considerar a capacidade de manutenção do disfarce.

O banco de vozes já havia sido utilizado em estudos anteriores. O texto, de 69 palavras, foi desenvolvido durante um trabalho de mestrado, por pesquisadoras da área de letras/linguística e de perícia, conforme segue: *“Alô, quero falar com a Dona Teca. Dona Teca, aqui fala é o capeta. Estamos com o pato do teu marido na toca e vamos matar ele, picar e tacar dentro de uma garrafa de Coca. Quer salvar ele? Então não me deixa puto. Quero um milhão. Bota tudo num pacote perto da pipa do cateto e se pica. Não chama a polícia senão te cutuco e espeto teu coco.”*

Pensando no uso durante situação de disfarce, foram contemplados fonemas diversos, sobretudo os oclusivos não vozeados [p, t, k] em contexto intervocálico, onset de sílabas tônicas, contexto fonológico anterior representado pela vogal [a]; e contexto fonológico posterior para [p]: [e, i, u]; para [t]: [a, e, ɛ, ɔ] e para [k]: [a, ɔ].

### Análise do disfarce

Para a análise dos tipos de disfarces, foram convidadas três juízas, especialistas em voz, com experiência superior a 15 anos em análise perceptivo-auditiva, inclusive com protocolos que envolvem análise de ajustes de trato vocal, como é o caso do Protocolo da Análise do Perfil Vocal de John Laver (1980). As análises procederam da seguinte forma: inicialmente, duas das três juízas analisaram as amostras de cada sujeito, aos pares, individualmente, levando-se em consideração a voz habitual do sujeito como *baseline*. Assim, as juízas deveriam apontar quais os recursos/ajustes utilizados pelo locutor na voz disfarçada quando esta era comparada com a voz habitual.

As pesquisadoras elaboraram um questionário contendo 21 opções de tipos de ajustes possíveis, baseando-se em adaptações comuns feitas pelas pessoas em situações de disfarce, de modo geral, sendo eles relacionados à modificações na qualidade da voz, frequência, intensidade, articulação, ressonância e modulação. As juízas deveriam assinalar um “x” quando a característica descrita foi utilizada pelo locutor durante a emissão da voz disfarçada.

As respostas que tiveram divergência entre as duas juízas foram enviadas à terceira juíza, para desempate e confirmação da utilização ou não do referido ajuste pelo falante. Para fins de concordância interna de cada juíza, houve repetição de 20% das amostras. O valor de Kappa obtido foi superior a 0,8 para todas elas, indicando concordância interna substancial.

Para a extração de dados de frequência fundamental foi utilizado o software de análise acústica PRAAT®, adotando-se os seguintes passos para todas as amostras de cada um dos participantes (habituais e disfarçadas): Importação do trecho completo de leitura; Análise da periodicidade do arquivo em relação ao *pitch* (f_o_), com ajuste dos parâmetros de acordo com o sexo do locutor; Geração de um arquivo específico para análise de f_o;_ Análise visual dos pontos de f_o_ do arquivo gerado para eliminação de pontos espúrios e artefatos; Extração das medidas de f_o_ do arquivo corrigido: média (soma dos valores obtidos no conjunto, ao longo de todo o trecho, dividindo-se pela quantidade de valores somados), mediana (valor que separa a metade maior e a metade menor de uma amostra), f_b_ (medida estatística que define a frequência para qual as pregas vocais retornam naturalmente após uma excursão prosódica)^(18,19)^, mínima (menor valor de f_o_ observado ao longo de um trecho) e máxima (maior valor de f_o_ observado ao longo de um trecho); Criação do gráfico de distribuição a partir do arquivo de *pitch*, verificando-se a distribuição de f_o_.

Esclarece-se, portanto, que a extração ocorreu conforme os seguintes passos: 1. Os limites de análise de pitch foram ajustados de acordo com o sexo (75 a 300 Hz masculino e 100a 500 Hz feminino); 2. Verificação do comportamento da curva de pitch gerada com os pulsos considerados automaticamente; 3. Os tempos de produção dos trechos de cada indivíduo não foram considerados, tendo em vista que os cálculos de f_o_ e f_b_ são medidos em Hz; 4. Criação de Objeto Pitch; 5. Comparação visual do oscilograma, pulsos e curva de Pitch do objeto Sound com os pontos (amostras) do Objeto Pitch. Exclusão de amostras que não fossem correspondentes a sinal de voz; 6. Obtenção dos valores de frequência do objeto Pitch corrigido; 7. Criação do gráfico de distribuição a partir da matriz do Objeto Pitch.

A análise descritiva dos resultados das variáveis quantitativas foi realizada por meio de frequência relativa e frequência absoluta. Todos os valores numéricos obtidos nos procedimentos descritos acima foram tabulados em planilha eletrônica.

Os valores de f_o_ média, mediana, mínima e máxima e de f_b_ de vozes habituais foram comparados com valores de f_o_ média, mediana, mínima e máxima e de f_b_ de vozes disfarçadas por meio de testes estatísticos para amostras dependentes. Assim, partiu-se do pressuposto de que a amostra em situação habitual era a amostra controle em relação à amostra em situação de disfarce.

### Análise estatística

Os testes utilizados foram: Teste T de Student Dependente e Teste de Wilcoxon. Foram utilizados, ainda, testes para amostras dependentes, para a comparação de f_o_ média, mediana, mínima e máxima f_b_, nos momentos de fala habitual e disfarçada, para homens e mulheres. Foram utilizados os testes T de Student Dependente ou Wilcoxon, a depender da distribuição de dados (normal ou anormal), analisada por meio do Teste de Normalidade de Shapiro Wilk. Diante de diferenças estatisticamente significantes, foi calculado o tamanho do efeito com d de Cohen (variáveis com diferença tiveram distribuição normal). A interpretação do tamanho do efeito se deu do seguinte modo: 0,20 efeito pequeno; 0,5 efeito médio; 0,8 efeito grande.

Para todas as análises adotou-se o nível de significância de 5% ou 0,05.

## RESULTADOS

Na Tabela 1 está apresentada a caracterização da amostra por meio de análise descritiva dos ajustes vocais utilizados pelos locutores ao realizarem as vozes disfarçadas em relação às vozes habituais (Tabela 1).

**Tabela 1:** Análise descritiva dos tipos de disfarces usados pelos falantes

**Variável**	**Amostra total (n=40)**		**Sexo feminino (n=20)**		**Sexo masculino (n=20)**	
	**n**	**%**	**n**	**%**	**n**	**%**
Voz tensa	21	52,50	9	45,00	12	30,00
Loudness mais forte	21	52,50	10	50,00	11	27,50
Pitch mais grave	18	45,00	9	45,00	9	45,00
Distorções /omissões /substituições fonêmicas	16	40,00	5	25,00	11	27,50
Pitch mais agudo	14	35,00	9	45,00	5	25,00
Constrição faríngea	14	35,00	4	20,00	10	50,00
Rugosidade/ Crepitação	12	30,00	3	15,00	9	45,00
Arredondamento/ Protrusão de lábios	8	20,00	5	25,00	3	15,00
Fechamento /travamento mandibular/ articulatório	6	15,00	2	10,00	4	20,00
Hipernasalidade	6	15,00	2	10,00	4	20,00
Estiramento de lábios	5	12,50	2	10,00	3	15,00
Falsete	4	10,00	1	5,00	2	10,00
Variações de pitch	3	7,50	3	15,00	0	0,00
Variações de loudness	3	7,50	0	0,00	0	0,00
Loudness mais fraca	2	5,00	1	5,00	1	5,00
Hiponasalidade	2	5,00	2	10,00	0	0,00
Abertura exagerada de mandíbula / sobrearticulação	2	5,00	1	5,00	1	5,00
Sussurro/ voz cochichada	1	2,50	1	5,00	0	0,00
Tremor vocal	1	2,50	0	0,00	1	5,00
Soprosidade	0	0,00	0	0,00	1	5,00
Voz relaxada	0	0,00	0	0,00	0	0,00

Observa-se que os recursos de voz tensa e *loudness* mais forte foram os mais utilizados (n=21;52,50%), seguido por rebaixamento do *pitch/pitch* mais grave (n=18;45%), distorções/omissões/substituições fonêmicas (n=16; 40%), *pitch* mais agudo e constrição faríngea (n=14;35% em ambos). Apesar de haver várias frequências relativas maiores em termos descritivos entre homens e mulheres, o teste de igualdade de proporções, realizado de forma complementar, indicou p>0,05 20 dos 21 cruzamentos entre os sexos, havendo diferença apenas para o uso da constrição faríngea, mais utilizada por homens (n=10;50%) do que mulheres (n=4;20%) - p=0,047.

Quanto à comparação entre os resultados referentes à frequência fundamental da voz (f_o_), os valores das medidas f_b_, f_o_ mínima e f_o_ máxima não apresentaram diferenças quando as duas situações foram comparadas, vozes disfarçadas e vozes habituais. Isso não foi observado para as medidas de f_o_ média (habitual 198,85 e em disfarce 216,62 - p = 0,021) e f_o_ mediana (habitual 195,31 e em disfarce 206,1 - p = 0,032), que apresentaram diferenças entre as emissões, com valores mais elevados na emissão de disfarce (Tabela 2). O cálculo adicional de d de Cohen indicou tamanho do efeito de pequeno a médio para as variáveis f_o_ média e f_o_ mediana.

**Tabela 2:** Comparação entre os resultados referentes à frequência fundamental da voz (f_o_) durante emissão habitual e emissão em situação de disfarce, no grupo total (n =40)

**Variável**	**Emissão habitual**					**Emissão em Disfarce (D)**					**p**	**Tamanho efeito**
	**Média**	**Mediana**	**Mínimo**	**Máximo**	**DP**	**Média**	**Mediana**	**Mínimo**	**Máximo**	**DP**		
f_o_ de base^***^	147,276	141,422	86,684	214,887	39,633	153,42	158,533	83,532	250,333	44,356	0,333	
f_o_ média^**^	198,857	200,22	109,132	299,64	56,289	216,624	210,061	120,428	365,543	59,607	**0,021** ^*^	0,38
f_o_ mediana**	195,31	198,842	106,322	286,391	56,09	211,87	206,106	112,624	367,332	62,018	0,032*	0,35
f_o_ mínima***	79,584	76,349	50,315	121,211	13,602	80,581	75,804	53,351	158,172	20,272	0,751	
f_o_ máxima***	485,904	579,378	179,868	639,778	144,06	486,715	553,68	195,081	609,516	126,846	0,968	

* p<0,05

** Teste T de Student Dependente e tamanho de efeito com d de Cohen

*** Teste de Wilcoxon

Legenda: DP = desvio padrão

Na comparação entre os resultados referentes à frequência fundamental da voz (f_o_) durante a emissão habitual e em situação de disfarce, não houve diferença para nenhuma das medidas analisadas, para o sexo feminino (Tabela 3).

**Tabela 3:** Comparação entre os resultados referentes à frequência fundamental da voz (f_o_) durante emissão habitual e emissão em situação de disfarce, no sexo feminino (n=20)

**Variável**	**Emissão habitual (H)**					**Emissão em Disfarce (D)**					**p**
	**Média**	**Mediana**	**Mínimo**	**Máximo**	**DP**	**Média**	**Mediana**	**Mínimo**	**Máximo**	**DP**	
f_o_ de base^***^	182,909	182,017	143,504	214,887	17,456	183,043	182,707	107,446	250,333	31,343	0,709
f_o_ média^**^	248,492	248,805	201,112	299,64	28,463	250,495	236,291	188,194	365,543	46,565	0,808
f_o_ mediana**	244,347	243,453	199,114	286,391	28,914	247,385	234,939	183,036	367,332	47,504	0,729
f_o_ mínima***	82,325	78,949	64,471	121,211	14,163	83,703	75,825	53,351	158,172	27,305	0,970
f_o_ máxima***	531,226	584,084	316,243	639,778	101,149	512,296	570,637	314,665	609,516	102,26	0,433

** Teste T de Student Dependente

*** Teste de Wilcoxon

Legenda: DP = desvio padrão

Por fim, a Tabela 4 refere-se à comparação entre os resultados referentes à frequência fundamental da voz durante emissão habitual e em situação de disfarce para o sexo masculino, nesta análise houve diferenças entre os resultados de f_o_ média (habitual 149,22 e em disfarce 182,75 - p = 0,09) e f_o_ mediana (habitual 146,27 e em disfarce 176,35 - p= 0,018). Nesta tabela, os tamanhos do efeito das variáveis f_o_ média e f_o_ mediana foram considerados de médios a grandes.

**Tabela 4:** Comparação entre os resultados referentes à frequência fundamental da voz (f_o_) durante emissão habitual e emissão em situação de disfarce, no sexo masculino (n=20)

Variável	Emissão habitual (H)					Emissão em Disfarce (D)					p	Tamanho do efeito
	Média	Mediana	Mínimo	Máximo	DP	Média	Mediana	Mínimo	Máximo	DP		
f_o_ de base^***^	111,643	114,4	86,684	139,339	15,697	123,796	118,027	83,532	228,158	34,765	0,108	
f_o_ média^**^	149,222	148,514	109,132	199,329	22,516	182,753	172,251	120,428	314,233	52,053	**0,009** ^*^	0,66
f_o_ mediana**	146,274	145,35	106,322	198,57	23,655	176,355	158,664	112,624	331,095	54,616	0,018*	0,58
f_o_ mínima***	76,844	75,484	50,315	96,274	12,781	77,458	75,804	65,208	102,531	8,799	0,737	
f_o_ máxima***	440,582	525,051	179,868	601,281	167,461	461,134	532,108	195,082	602,068	145,574	0,332	

* p<0,05

** Teste T de Student Dependente e tamanho de efeito com d de Cohen

*** Teste de Wilcoxon

Legenda: DP = desvio padrão

## DISCUSSÃO

Durante a emissão da fala há modificações de *pitch*, para grave e agudo, pois ele é o elemento responsável pela entonação (variação de frequência do ponto de vista da percepção psicoacústica)^(15)^. Na emissão da fala, o emissor produz uma variação de *pitch* por consequência dos diversos tons usados para se expressar. Nesse cenário, a análise acústica da frequência fundamental permite o traçado de gráficos chamados de *curvas de contorno de pitch*, nos quais encontra-se valores instantâneos da frequência fundamental em função do tempo^(29)^.

Os resultados obtidos neste estudo reforçam estudo anterior^(15)^ a respeito das comuns variações de *pitch* em situações de disfarce, uma vez que o uso do pitch mais grave foi o segundo recurso mais usado, aparecendo em 45% dos disfarces da população geral e também da população feminina e masculina. Além disso, o *pitch* mais agudo também apareceu entre um dos disfarces preferidos pelos locutores, com 32,5% na população geral. As variações de *pitch* ocorreram em 7,5% dos casos de disfarce desse estudo no grupo geral, aparecendo apenas na população feminina, em 15% dos disfarces.

Em estudo recente^(30)^, os autores propuseram analisar os efeitos do disfarce na frequência fundamental da voz e se esses afetaram ou não as análises acústicas. Os autores observaram que um dos tipos de ajustes mais usados foi a constrição do trato, que indicou forte associação nos valores de f_o_ de disfarce no controle das amostras de homens (p=0,105) e em mulheres (p=0,171). Com isso, pode-se entender que mesmo com a constrição do trato presente, é possível realizar as análises acústicas e constatar se trata-se ou não do mesmo sujeito na locução.

Outro tipo de disfarce presente no estudo é a simulação da raiva pelos participantes^(30)^. No presente estudo as variações que a raiva pode trazer para a produção do disfarce também aparecem se traduzindo como *loudness* mais forte, sendo este o recurso mais usado pelo grupo total. Todos os recursos supracitados acerca do estudo anterior^(30)^ indicaram forte associação nos valores de f_o_ do disfarce com o controle nas amostras de homens e mulheres. No referido estudo, o gênero feminino apresentou maiores variações nos valores de f_o_ das amostras disfarçadas em comparação ao gênero masculino, resultado este que é divergente do presente estudo, no qual as mulheres fizeram alterações menores na f_o_ do que os homens.

Quando se trata de processos legais, a opinião de especialistas vem sendo cada vez mais requisitada e inserida nesses processos, com o intuito de responder se determinada amostra de voz pertence ou não ao mesmo falante^(3)^. Essa comparação amostral e seus respectivos resultados correspondem ao que se denomina “Identificação Forense do Falante”^(4)^. No presente estudo foi possível verificar que algumas medidas acústicas são mais estáveis na comparação de amostras habituais e em situação de disfarce de um mesmo indivíduo, o que pôde ser confirmado pela não ocorrência de diferenças estatisticamente significantes. Este fato é evidenciado quando se observa que as medidas f_b_, f_o_ máxima e f_o_ mínima mostraram resultados semelhantes em ambas as amostras apesar dos variados ajustes de trato vocal realizados pelos locutores. Tais resultados refletem uma não suscetibilidade de mudança dessas medidas mediante as modificações realizadas no trato vocal. Tal fato pode contribuir para o laudo pericial afirmando ou refutando a hipótese de se tratar do mesmo falante no possível crime em questão (Tabela 2).

Entendendo, a partir desses resultados, que f_b_, f_o_ máxima e f_o_ mínima se alteram menos do que as demais frequências, podemos fazer um paralelo com um estudo^(11)^ em que as análises estatísticas comprovaram que, quando se simulavam diferentes tipos de disfarces através da variação em relação ao estilo de fala/diferentes emoções, esforço vocal e qualidade de gravação, existem diferenças nas medidas de frequência fundamental média e mediana quando o locutor é comparado com ele mesmo. O que os autores referem neste estudo^(16)^ é que os resultados para f_o_ média, f_o_ mediana e desvio padrão foram afetados por *outliers -* termo utilizado dentro da estatística para se referir a achados que fogem à normalidade, são atípicos e podem causar prejuízos à interpretação dos resultados das amostras em questão. Reforça-se que no presente estudo tais *outliers* foram retirados a fim de minimizar este viés.

No presente estudo, os resultados obtidos através do f_b,_ f_o_ máxima e f_o_ mínima foram semelhantes nas situações de fala habitual e fala em disfarce, apesar dos variados recursos utilizados pelos locutores. A f_b_ é a frequência de vibração das cordas vocais em uma posição relaxada, a frequência para qual as pregas vocais retornam naturalmente após uma excursão prosódica^(19)^. Assim, hipotetiza-se que ela seja menos suscetível a variações diversas de fala tanto do ponto de vista prosódico quanto de modificações de órgãos fonoarticulatórios, o que pode contribuir para o laudo pericial na análise de um crime quanto à comparação de falantes.

Até o momento, a análise forense da frequência fundamental tem-se concentrado em sua média de duração e desvio padrão^(15)^. A partir da análise dos resultados do presente estudo, é possível sugerir que a média de duração, assim como a mediana de duração podem ser medidas menos estáveis para a comparação de amostras e em situação de disfarce, pois apresentam alterações estatisticamente significantes. A média da frequência fundamental (f_o_) pode ser bastante influenciada por fatores externos, como por exemplo, as suscetíveis emoções^(19)^. Talvez isso possa hipotetizar o fato de f_o_ média e mediana terem tido diferenças entre as emissões habituais e disfarçadas. É importante mencionar que, no grupo geral, os tamanhos do efeito em relação às comparações de fala habitual e disfarçada em relação à f_o_ média e mediana foram considerados de pequenos a médios, ou seja, são efeitos visíveis e que podem ser relevantes nos contextos clínicos e/ou de periciais. Já em relação exclusivamente ao sexo masculino, os tamanhos do efeito nessas mesmas medidas foram considerados de médios a grandes, podendo-se inferir que há grande relevância prática nas diferenças encontradas.

São diversos os fatores que influenciam e afetam a frequência fundamental, sendo esses fatores divididos didaticamente como fatores técnicos, fisiológicos e fatores psicológicos. Os fatores fisiológicos são as causas biológicas, como a idade e o tabagismo, já os fatores psicológicos estão ligados às emoções e condições ambientais no momento da emissão. Quanto aos fatores técnicos, esses dizem respeito às condições de gravação das amostras em questão^(31)^. Apesar dessas condições, a frequência fundamental (f_o_) é um parâmetro fonético com bons resultados e, por isso, considerado de confiança para análises forenses^(10)^. Entendendo que a estabilidade é um recurso chave na perícia forense para os parâmetros de comparação de falantes, a frequência fundamental parece atender uma vasta gama desses critérios^(31)^. Com essa referida estabilidade da frequência fundamental em vista, o presente estudo abrangeu outras medidas dentro da f_o,_ fortalecendo a ideia citada anteriormente^(16,17)^ de que a frequência fundamental de base (f_b_) se mostra mais estável em relação a outros parâmetros de frequência fundamental como, por exemplo, as já citadas f_o_ média e f_o_ mediana.

Partindo desses saberes, fica claro que a frequência fundamental de base (f_b_) pode ser usada na comparação de locutores pela perícia, em situações de disfarce, juntamente com a f_o_ máxima e f_o_ mínima, que trouxeram resultados confiáveis na análise pericial em casos de crimes ou suspeita de crime.

Quanto às limitações deste estudo, não se avaliou a variabilidade natural da f_o_ de um mesmo falante em situações não disfarçadas. Isso poderia contribuir para a avaliação do quanto os efeitos observados neste estudo foram atribuíveis ao disfarce vocal ou a flutuações da emissão vocal, sendo esta análise uma possibilidade para estudos futuros.

Considera-se que novas pesquisas possam trabalhar com amostras de frases e falas espontâneas ou semiespontânea, além da leitura de um texto normalmente e com disfarce, para a obtenção de uma estimativa/valor de corte, em Hz, de uma medida para outra podendo, futuramente, traçar um parâmetro de Hz aceitável para que se saiba se aquelas amostras são consideradas ou não do mesmo sujeito, tornando essas inferências mais aprofundadas.

Estudos cujos delineamentos metodológicos contemplem métricas de acurácia, sensibilidade e especificidade poderão permitir identificações entre diferentes indivíduos/falantes, para além das comparações intraindividuais realizadas na presente pesquisa.

## CONCLUSÃO

A partir dos resultados obtidos, conclui-se que, as medidas de f_b_, f_o_ mínima e f_o_ máxima mostraram maior estabilidade frente à situação de disfarce, o que pode sugerir utilidade futura em comparações intraindividuais. Diante do exposto, a f_b_, medida ainda pouco explorada e difundida dentro da perícia criminal, mostra-se útil para a análise de comparação de locutores, auxiliando na produção do laudo pericial. Os variados tipos de ajustes feitos pelos locutores para a realização dos disfarces não alteraram de modo significativo as medidas de f_b_, f_o_ máxima e f_o_ mínima quando as situações de fala habitual e falas disfarçadas foram comparadas.
